# Single-Shot 3D Multi-Person Shape Reconstruction from a Single RGB Image

**DOI:** 10.3390/e22080806

**Published:** 2020-07-23

**Authors:** Seong Hyun Kim, Ju Yong Chang

**Affiliations:** Department of Electronics and Communication Engineering, Kwangwoon University, Seoul 01897, Korea; thuthdew15@kw.ac.kr

**Keywords:** 3D human shape reconstruction, statistical body shape model, deep neural network

## Abstract

Although the performance of the 3D human shape reconstruction method has improved considerably in recent years, most methods focus on a single person, reconstruct a root-relative 3D shape, and rely on ground-truth information about the absolute depth to convert the reconstruction result to the camera coordinate system. In this paper, we propose an end-to-end learning-based model for single-shot, 3D, multi-person shape reconstruction in the camera coordinate system from a single RGB image. Our network produces output tensors divided into grid cells to reconstruct the 3D shapes of multiple persons in a single-shot manner, where each grid cell contains information about the subject. Moreover, our network predicts the absolute position of the root joint while reconstructing the root-relative 3D shape, which enables reconstructing the 3D shapes of multiple persons in the camera coordinate system. The proposed network can be learned in an end-to-end manner and process images at about 37 fps to perform the 3D multi-person shape reconstruction task in real time.

## 1. Introduction

In recent years, 3D human shape reconstruction from a single RGB image has been actively studied as one of the challenging tasks of computer vision, but most studies address the case of a single person. Most recent methods [1,2,3,4,5] for single-person shape reconstruction regress the parameters of a statistical body shape model, such as the skinned multi-person linear (SMPL) model [6], while using a deep neural network to reconstruct a 3D shape.

Most 3D human shape reconstruction methods focus on a single person, but real-world applications require processing multiple persons in real time. Leo et al. [7] proposed a system that can detect multiple people’s interactions in a crowded sports scene in real-time. Moon et al. [8] proposed a pose refinement network that outputs a refined 2D pose from an input pair of an RGB image and its corresponding noisy 2D pose, which can be used for top-down 2D multi-person pose estimation. However, this method generates 2D image coordinates of human joints, which only provide sparse information about the target human subject. On the other hand, our goal in this paper is to obtain dense 3D shapes that can provide richer information about multiple people from an input single RGB image, that is, 3D multi-person shape reconstruction. Additionally, the problem we address in this paper is not related to the pose refinement in [8].

However, the goal of this paper, the 3D multi-person shape reconstruction problem, has been less studied than the single-person case. The existing multi-person shape reconstruction method in [9] reconstructs the 3D shapes of all persons from an input image in a bottom-up manner. In general, the bottom-up approach runs faster than the top-down method, but in the case of [9], the optimization based on binary integer programming requires a lot of time, which prevents the 3D multi-person shape reconstruction task from being processed in real time. Moreover, most methods estimate a root-relative 3D pose or shape, and the acquisition of pose or shape in the camera coordinate system requires ground-truth absolute depth information. Moon et al. [10] recently proposed a new method to predict the absolute depth of the root joint to solve this problem and consequently estimate the 3D poses of multiple persons in the camera coordinate system. However, the method of [10] focuses on pose estimation rather than shape reconstruction, and the entire system cannot be learned in end-to-end fashion due to the separation of pose estimation and absolute depth estimation modules.

**Figure 1:**
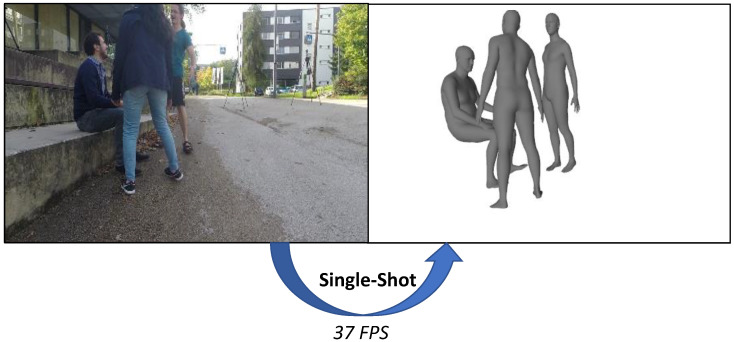
Given a single red, green, and blue (RGB) image, our goal is to reconstruct the 3D shapes of multiple persons in a single-shot fashion. Our single-shot method can process images at about 37 frames per second.

In this paper, we propose an end-to-end learning-based model for single-shot 3D multi-person shape reconstruction in the camera coordinate system from a single RGB image as in Figure 1. Our framework outputs a tensor of grid structure in a single-shot manner, where each grid cell contains information about the person contained therein. Moreover, we use the distance computation method proposed in [10] to predict the absolute depth of a person in the camera coordinate system. This approach requires bounding box information tightly enclosing a person, but we assume that no bounding box information is given as input considering the single-shot approach. Instead, we designed the network to predict additional bounding box information internally. Unlike previous top-down methods, our single-shot bottom-up method works in real time with constant complexity, regardless of the number of persons in an input image. In experiments, we performed quantitative and runtime comparisons with the baseline method and proved that the proposed method achieves comparable performance and has advantages in terms of runtime. Moreover, herein we show through comparisons that the proposed method outperforms many state-of-the-art methods in terms of runtime and quantitative performance.

In summary, the key contributions in this paper are as follows. First, the proposed single-shot network processes images at about 37 fps to perform the 3D multi-person shape reconstruction task in real-time. Second, our method predicts not only the 3D shape but also the depth information of the root joint, and as a result, reconstructs the 3D shapes of multiple persons based on the camera coordinate system.

The rest of this paper is organized as follows. Section 2 introduces existing studies on human pose estimation and human shape reconstruction. Section 3 presents the proposed single-shot, 3D, multi-person shape reconstruction method. Section 4 quantitatively and qualitatively evaluates the performance of the proposed method and compares it with the baseline and existing state-of-the-art methods. Finally, Section 5 concludes this paper.

## 2. Related Works

### 2.1. 2D Multi-Person Pose Estimation

The 2D multi-person pose estimation methods are broadly divided into two categories: (1) top-down methods and (2) bottom-up methods. A top-down method first detects all persons in the image using a human detector, and then crops each detected person into a bounding box and feeds him/her into a single-person pose estimator. By contrast, a bottom-up method first predicts all joints in the input image and then assigns the predicted joints to each person through clustering.

The works [11,12,13,14] belong to the top-down method type. Papandreou et al. [13] proposed a method that predicts 2D offset vectors and 2D heatmaps, and then fuses them to generate refined heatmaps. Xiao et al. [14] proposed a simple network consisting of a deep backbone network and several upsampling layers. Chen et al. [11] proposed a cascaded pyramid network that focuses on hard keypoints to refine the initially estimated pose. Moon et al. [12] proposed a multi-scale aggregation R-CNN network that performs human detection and keypoint localization simultaneously in a single model.

The works [15,16,17,18] belong to the bottom-up method type. Cao et al. [15] proposed part affinity fields (PAFs) that model the relations between human keypoints as 2D vectors. Newell et al. [18] proposed a network that predicts similar tag values for keypoints belonging to the same person and different tag values for keypoints belonging to different persons to assign predicted joints to a specific person. Kocabas et al. [16] proposed a pose residual network that assigns predicted joints to a specific person. Kreiss et al. [17] proposed the PifPaf that combines a part intensity field (PIF) for predicting human joints and a part association field (PAF) for assigning predicted joints to each pose.

### 2.2. 3D Multi-Person Pose Estimation

The 3D multi-person pose estimation problem has been studied less than the 2D multi-person case. Rogez et al. [19] proposed a top-down method called Lcr-net, which consists of localization, classification, and regression parts. The network generates human pose proposals, and then classifies the generated human poses into several anchor poses, and refines the poses through regression. Moon et al. [10] proposed a camera-distance-aware top-down method. Their network consists of PoseNet, which predicts root-relative 3D poses, and RootNet, which predicts the absolute 3D pose of the root joint. They combined the outputs of PoseNet and RootNet to generate the final absolute 3D pose in the camera coordinate system. Mehta et al. [20] proposed a bottom-up 3D multi-person pose estimation network that predicts an occlusion-robust pose map and PAFs [15].

### 2.3. 3D Human Shape Reconstruction

Traditional methods [21,22,23] optimize the objective function to fit the parametric body model to manually annotated silhouettes or 2D keypoints. Bogo et al. [24] proposed an optimization-based method called SMPLify that fits the parametric body model to 2D keypoints extracted using the off-the-shelf keypoint detector [25] to automate the manual annotation. SMPLify can automatically reconstruct the 3D shape from a single RGB image, but its disadvantage is that the optimization to fit the parametric body model to 2D keypoints takes about 20–60 s per image.

Regression-based methods using deep neural networks have been recently proposed to overcome the shortcomings of the previous methods. Kanazawa et al. [1] proposed a method to minimize the 2D projection error between the 2D projection of the 3D joint obtained from the predicted SMPL parameters and the ground-truth 2D joint. The proposed model is trained to generate SMPL parameters that correspond to the valid 3D shape of a human using a discriminator network because the 2D projection loss alone does not provide sufficient constraints. Varol et al. [26] exploited a volumetric representation and reconstructed the 3D human shape through volumetric regression. Pavlakos et al. [3] predicted SMPL parameters using keypoints and silhouettes as intermediate representations. Kolotouros et al. [27] proposed a method that combines the feedforward regression step and the SMPLify-based optimization step into a loop structure to merge the advantages of a regression-based method and an optimization-based method. In addition to the human body, methods for 3D reconstruction of the human hand and face have recently been proposed. The newly proposed method of Wu et al. [28] reconstructs the 3D shape of a human face based on unsupervised learning.

All these deep learning-based methods reconstruct the 3D shape of a single person. The 3D multi-person shape reconstruction problem has been studied less than the single-person case. Zanfir et al. [9] reconstructed the 3D shapes of multiple people in a bottom-up fashion. They proposed a limb-scoring network to predict the connection likelihood between joints and groups the joints using binary integer programming. Their method is a bottom-up approach, but its disadvantage is that it cannot process the multi-person 3D shape reconstruction task in real time because of the high computational complexity of the optimization using binary integer programming. In this paper, we propose an end-to-end learning-based model for single-shot, 3D, multi-person shape reconstruction in the camera coordinate system from a single RGB image.

## 3. Proposed Methods

**Figure 2:**
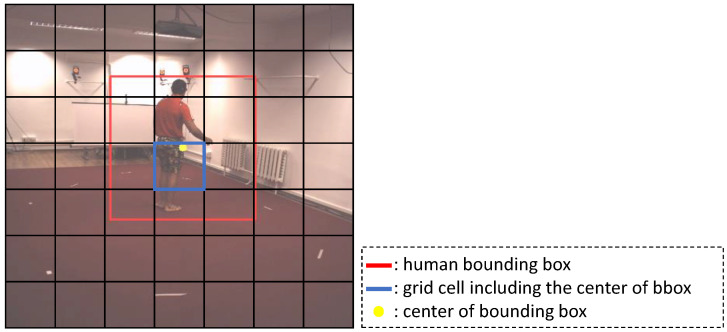
An example of a grid representation.

Our goal is to reconstruct the 3D shapes of multiple persons based on the camera coordinate system from a single RGB image in a single-shot manner. We were inspired from the ideas of the existing single-step object detection methods [29,30,31] to reconstruct the 3D shapes of multiple persons in a single-shot manner. Similarly to those methods, our network produces an output tensor divided by a grid of size H×W. Figure 2 shows an example of a grid representation used in this paper. If the center (yellow dot) of the human bounding box (red rectangle) is located within a grid cell (blue rectangle), then the network should predict that the grid cell contains a subject.

**Figure 3:**
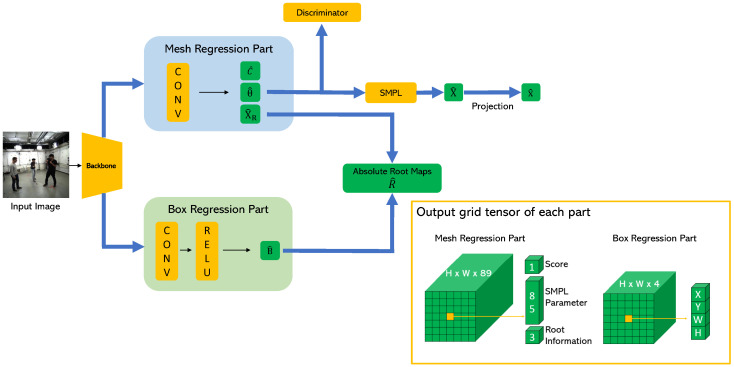
Overview of the proposed network. First, the backbone network extracts the convolutional features from the input image. The extracted features are fed into the mesh regression part (MRP) and box regression part (BRP) simultaneously and the skinned multi-person linear (SMPL) model parameters $\hat{\theta }$ estimated from the MRP are fed into the discriminator network that determines whether the predicted SMPL parameters correspond to real human bodies or not. The absolute depth of the root joint is obtained using the outputs of the MRP and BRP.

Figure 3 shows the structure of the proposed network that can be trained in an end-to-end fashion. First, the backbone network extracts the convolutional features from the input single RGB image. Then, the extracted features are simultaneously fed into the mesh regression part (MRP) and the box regression part (BRP). The outputs of the MRP are as follows: (1) score maps $\hat{C}\in R^{H\times W}$ to indicate the probability that each grid cell will contain the subject; (2) mesh maps $\hat{\theta }\in R^{H\times W\times 85}$ that contain the pose, shape, and camera parameters of the SMPL model; and (3) root maps ${\hat{X}}_{R}\in R^{H\times W\times 3}$ including the root joint information, where the first two channels represent offset from the top-left corner of the grid cell where the center of the bounding box is located to the root joint, and the last channel represents the correction factor γ which refines the estimated camera distance as in [10]. The predicted mesh maps $\hat{\theta }$ are fed into the adversarial discriminator network, which determines whether the estimated parameters are real using the large dataset of 3D human meshes [32]. The discriminator network allows the SMPL parameter in the grid cell where the subject is located to generate a valid 3D human shape and weakly supervises in-the-wild images without the 3D ground-truth. Moreover, we assume that no bounding box information is given as input because the proposed method reconstructs the 3D shape in a single-shot fashion. Therefore, we let the network predict the bounding boxes additionally to calculate the camera distance of the root joint. The output of the BRP is as follows: bounding box maps $\hat{B}\in R^{H\times W\times 4}$, where the first two channels represent the offset from the top-left corner of the grid cell where the center of the bounding box is located to the center of the bounding box, and the other two channels represent the width and height of the bounding box.

### 3.1. 3D Body Representation

We use the SMPL [6] to represent a 3D human mesh. Shape $\beta \in R^{10}$ is parameterized by the first 10 coefficients in the PCA shape space. Pose $\theta \in R^{72}$ is parameterized by the relative 3D rotations of the 23 joints and the three global body rotations in the axis-angle representation. The SMPL model provides a differentiable function that outputs body mesh $M\left(\theta ,\beta \right)\in R^{3\times N}$ (*N* = 6890) from pose θ and shape β parameters. The 3D joint $X\in R^{3\times J}$ is calculated using a pre-trained linear regressor $W\in R^{N\times J}$ as follows:

(1) X(θ,β)=M(θ,β)W,

where *J* denotes the number of joints.

We project the 3D joints into the 2D image plane using the weak-perspective camera model that includes scale s∈R and translation $t\in R^{2}$ parameters. The equation for projecting 3D joints *X* into 2D joints $x\in R^{2\times J}$ using the camera parameters *s*, *t* is as follows:

(2) $$x_{i}=s\Pi \left(RX_{i}\left(\theta ,\beta \right)\right)+t,$$

where Π, $R\in R^{3\times 3}$, and subscript *i* denote the orthographic projection, the global body rotation, and the index of column vector, respectively.

The notation used in this subsection is for a single person to describe the SMPL model. In the rest of the paper except for this subsection, we use grid-style notation.

### 3.2. Box Regression Part

We estimate the absolute depth of the root joint to reconstruct the 3D human shape in the camera coordinate system. The naive approach to predict the depth of the root joint is to regress the depth directly from the input image. However, the input image implicitly provides the relative locations of subjects contained in the image through appearance information but does not provide clues about the absolute depth, such as camera information, which makes directly regressing the absolute depth from the input image alone difficult for the network. To alleviate this problem, we use the distance measure proposed in [10] to obtain the absolute depth. Distance measure *k* is as follows:

(3) $$k=\sqrt{f_{x}f_{y}\frac{A_{real}}{A_{img}}},$$

where $f_{x}$ and $f_{y}$ are the focal lengths, and $A_{real}$ and $A_{img}$ denote the areas of a person in real space $\left(mm^{2}\right)$ and image space $\left(pixel^{2}\right)$, respectively. This distance measure approximates the absolute depth from the camera to the subject using the camera’s focal lengths and the ratio between the subject’s areas in real space and image space. We assume that the focal length is given in the dataset and set $A_{real}$ as 2000 mm × 2000 mm considering the case of an adult. Equation (3) can be easily derived by a pinhole camera projection model.

The absolute depth estimation method in [10] is a top-down approach, so it assumes a ground-truth, or an estimated bounding box is given to calculate the area of a person in the image. By contrast, we assume that bounding box information is not available because we seek the single-shot method. Therefore, we add BRP, a module that generates bounding box information, to the proposed network. We also allow BRP and MRP to share the same backbone network and thus train the proposed network in an end-to-end manner.

After the convolutional features of the backbone network are fed into the BRP, the BRP predicts the bounding box maps $\hat{B}\in R^{H\times W\times 4}$. Each grid cell of the bounding box maps contains vectorized bounding box information. The loss function for bounding box maps is as follows:

(4) $$L_{bbox}=\sum_{h}^{H}\sum_{w}^{W}C_{h,w}{∥{\hat{B}}_{h,w}-B_{h,w}∥}_{2}^{2},$$

where *h* and *w* are the indices of the grid, *B* represents the ground-truth bounding box maps, and *C* represents the ground-truth score maps. $C_{h,w}$ acts as a weight that makes the loss value 0 for a grid cell that does not contain a subject.

We can derive the absolute depth *k* through Equation (3) by calculating area $A_{img}$ of the subject in the image from the width and height of the predicted $\hat{B}$. We express *k* using the grid representation, which we call distance measure maps $\hat{D}\in R^{H\times W}$. Each grid cell of distance measure maps contains a *k* value calculated from the bounding box corresponding to that grid cell. However, the person’s actual body shape and posture cannot be reflected, which results in inaccurate results, because the absolute depth calculated by Equation (3) fixes a person’s area in real space to 2000 mm × 2000 mm. Therefore, we modify the incorrect *k* value through the element-wise product between γ and $\hat{D}$, and use this modified result as the final absolute depth of the root joint.

### 3.3. Mesh Regression Part

After the convolutional features of the backbone network are fed into the MRP, the MRP predicts the score maps, mesh maps, and root maps.

Each grid cell of the estimated score maps $\hat{C}\in R^{H\times W}$ contains the probability that the grid cell contains the subject. The loss function for the score maps is as follows:

(5) $$L_{score}=\sum_{h}^{H}\sum_{w}^{W}{\left({\hat{C}}_{h,w}-C_{h,w}\right)}^{2},$$

where *C* represents the ground-truth score maps.

Each grid cell in mesh maps $\hat{\theta }\in R^{H\times W\times 85}$ contains SMPL parameters that are vectorized. Our goal is to ensure that mesh maps estimate the correct SMPL parameters for the subject in the image. However, constructing a dataset that contains ground-truth SMPL parameters is generally very difficult. Therefore, we supervise the SMPL parameters implicitly by applying losses to the estimated 3D joints and the 2D joints projected from the 3D joints. We obtain the 3D joint coordinates from the predicted SMPL parameters through Equation (1) and apply the following loss:

(6) $$L_{3D}=𝟙\sum_{h}^{H}\sum_{w}^{W}\sum_{j}^{J}{∥{\hat{X}}_{h,w,j}-X_{h,w,j}∥}_{2}^{2},$$

where $X\in R^{H\times W\times J\times 3}$ represents the grid maps containing the ground-truth 3D joints and 𝟙 is an indicator function to make the loss value 0 for datasets which do not include the ground-truth 3D annotation.

For datasets with no ground-truth 3D joint annotation, we project the 3D joint obtained through the SMPL model into the 2D image plane and apply the loss function to the projected 2D joints. We project the 3D joints to the 2D joints through Equation (2) using the camera parameters of mesh maps. The 2D reprojection loss function is as follows:

(7) $$L_{repro}=\sum_{h}^{H}\sum_{w}^{W}\sum_{j}^{J}u_{h,w,j}{∥{\hat{x}}_{h,w,j}-x_{h,w,j}∥}_{2}^{2},$$

where $x\in R^{H\times W\times J\times 2}$ represents the grid maps containing the ground-truth 2D joints, and $u\in R^{H\times W\times J}$ represents the visibility maps for the joints (1 if visible; 0 otherwise).

The network predicts the 3D pose that can explain the projected 2D pose by minimizing the 2D reprojection loss. However, many 3D poses can explain the 2D pose, and most of them are anthropometrically implausible, causing the 3D shape produced by the network to be considerably different from the real human shape. Thus, we further train the discriminator network to make the SMPL parameters in grid cells containing the human subjects in the predicted mesh maps generate valid 3D human shapes. We adopt the adversarial training approach proposed in [1]. The 3D human mesh dataset annotated with SMPL parameters and the SMPL parameters predicted by the proposed network are used as real samples and fake samples for discriminator learning, respectively. As a result, the discriminator network determines whether the input SMPL parameters correspond to the shape of a real person or not. Following Kanazawa et al. [1], we decompose the pose and shape parameters of SMPL, and independently train two discriminator networks corresponding to the pose and shape parameters. The adversarial loss function for the proposed network is as follows:

(8) $$L_{adv}=E_{\theta ∽p_{E}}\left[{\left(D\left(\hat{\theta }\right)-1\right)}^{2}\right],$$

where *E* and *D* denote the proposed and discriminator networks, respectively. The loss function for the discriminator network is as follows:

(9) $$L_{dis}=E_{\theta ∽p_{data}}\left[{\left(D\left(\theta \right)-1\right)}^{2}\right]+E_{\theta ∽p_{E}}\left[D{\left(\hat{\theta }\right)}^{2}\right],$$

where $p_{data}$ denotes the 3D human mesh dataset [32] annotated with SMPL parameters.

Each grid cell of root maps ${\hat{X}}_{R}\in R^{H\times W\times 3}$ contains vectorized information related to the root joint. As mentioned in Section 3.2, the element-wise product between the correction factor γ corresponding to the third channel of root maps ${\hat{X}}_{R}$ and distance measure maps $\hat{D}$ is considered the refined absolute depth of root joint. We construct the absolute root maps $\hat{R}\in R^{H\times W\times 3}$ by replacing the last channel of the root maps with the refined absolute depth of the root joint, that is, the first two channels of $\hat{R}$ are the same as ${\hat{X}}_{R}$, and the last one contains the absolute depth refined by the correction factor γ. The loss function for absolute root maps $\hat{R}$ is as follows:

(10) $$L_{root}=\sum_{h}^{H}\sum_{w}^{W}{∥v_{h,w}⊙\left({\hat{R}}_{h,w}-R_{h,w}\right)∥}_{2}^{2},$$

where $\hat{R}$ represents the absolute root maps described above, and *R* is the corresponding ground-truth. The first two channels of $v\in R^{H\times W\times 3}$ denote the visibility of the root joint (1 if visible; 0 otherwise), and the last channel denotes the presence or absence of the 3D annotation.

The final loss function of the network is as follows:

(11) $$\begin{array}{cc} L_{total} & =\lambda_{b}L_{bbox}+\lambda_{s}L_{score}+\lambda_{r}L_{repro}+\lambda_{3D}L_{3D}\\ \; & +\lambda_{a}L_{adv}+\lambda_{root}L_{root}, \end{array}$$

where $\lambda_{b},\lambda_{s},\lambda_{r},\lambda_{3D},\lambda_{a},$ and $\lambda_{root}$ are the weighting factors that control the strength of each loss, and are set to $10^{3},10^{3},10,1,10^{3},$ and 1, respectively, for all experiments in this paper.

### 3.4. Implementation Details

We removed the last two layers (avgpool layer and softmax layer) of the pre-trained ResNet-50 [33] network for ImageNet classification, and used it as the backbone network to extract the convolutional features from an input RGB image. Extracted features were fed into the MRP and BRP simultaneously. The MRP consists of a single convolutional layer that outputs 89 (1+85+3) channels. If we allow the height or width of the output bounding box from BRP to be negative, then the absolute depth of the final root joint can also be negative, which makes training of the overall network difficult. Therefore, we constructed the BRP by combining one convolutional layer that outputs four channels (x, y, w, and h) and the ReLU activation layer to prevent negative output. The discriminator network has the same structure as the discriminator network proposed in [1]. We used 448×448 size images as input to the network, and the final outputs of the network were 14×14 tensors. We used the Adam optimizer [34] to optimize the loss function and set all learning rates to $1\times 10^{-4}$. We set the mini-batch size to 10, repeated the training total 1,050,000 iterations for sufficient convergence, and reduced the learning rates to $1\times 10^{-5}$ and $1\times 10^{-6}$ at 350,000 and 700,000 iterations, respectively. We implemented the proposed network based on the Pytorch [35] deep learning framework. All of our experiments were conducted in an environment with an Intel i7-7700K 4.2GHz CPU, 16GB RAM, and one Nvidia GTX1080Ti GPU.

In testing, we did not use any post-processing except non-maximum suppression (NMS). Using NMS, grid cells containing the subject are detected in score maps, and the root-relative 3D shape and the absolute depth of the root joint are reconstructed for detected subjects. Finally, these results are combined to reconstruct the 3D shapes of multiple persons based on the camera coordinate system. Algorithm 1 shows the overall procedure of reconstructing the shapes of multiple persons from an input RGB image.
**Algorithm 1** Procedure of obtaining the shapes of multiple persons from an input RGB image.**Input:** Single RGB Image *I*
**Output:** List of human body vertices *V*
1:$\hat{C},\hat{\theta },\hat{R}=Net\left(I\right)$                ▹ estimate tensors using the proposed network2:$K=NMS(\hat{C})$         ▹ perform NMS to obtain grid cells where the person is located3:V={}4:**for**k=1**to**Length(K)**do**5: h,w=K(k)6: $v=SMPL(\hat{\theta }\left(h,w\right))$         ▹ obtain human body vertices from the SMPL model7: $r=\hat{R}\left(h,w\right)$                     ▹ obtain the coordinates of the root joint8: r[:2]=PixelToCam(r[:2]) ▹ convert x,y coordinate of the root joint to the camera coordinate9: append v+r to *V*10:**end for**11:**return***V*

## 4. Experimental Results

**Figure 4:**
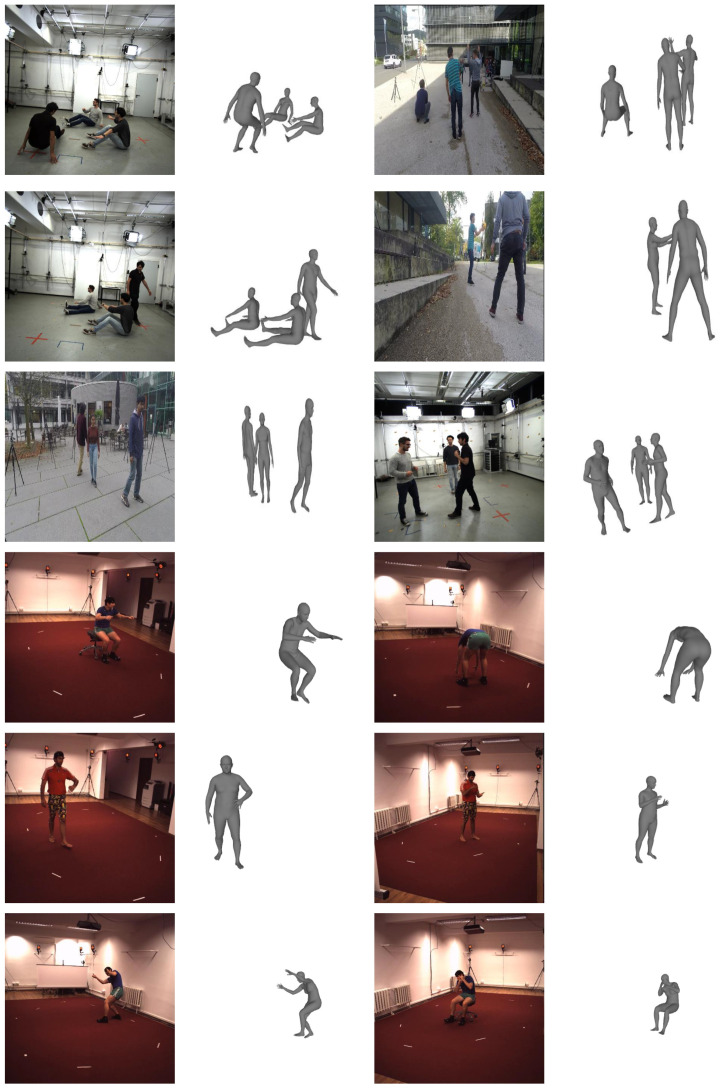
Qualitative results for various datasets: MuPoTS-3D (rows 1–3) and Human3.6M (rows 4–6).

We experimentally evaluated the proposed method. First, we describe the datasets and evaluation metrics used in training and evaluation. Next, we provide quantitative evaluation results and runtime results. We also provide qualitative evaluation results through Figure 4. Code and pretrained models are available at https://github.com/seonghyunkim1212/S2MPMR.

### 4.1. Datasets and Evaluation Metrics

Human3.6M dataset [36] is the largest 3D single-person pose dataset. This dataset consists of 3.6M video frames and provides images of actors performing various actions from four camera viewpoints. The ground-truth was obtained using a motion capture system, and seven (S1, S5, S6, S7, S8, S9, and S11) of the 11 subjects are annotated with the ground-truth 3D pose. We sampled videos every 50 frames and used five subjects (S1, S5, S6, S7, and S8) for training and two subjects (S9 and S11) for testing. When training, we used the additional 2D pose estimation dataset MPII [37] for generalization, and each mini-batch consisted of half Human3.6M and half MPII data. For MPII data, the indicator function of $L_{3D}$ and the weight for the absolute depth of $L_{root}$ were set to 0 because the MPII dataset has no 3D annotation. We used three evaluation metrics for the Human3.6M dataset. The first metric was the mean per joint position error (MPJPE) [36], which was widely used in previous works. MPJPE is calculated by first translating the root joints of the predicted pose and ground-truth pose to the origin, and then measuring the average Euclidean distance between the corresponding joints. The equation for calculating MPJPE is as follows:

(12) $$MPJPE=\frac{1}{J}\sum_{j=1}^{J}{∥{\hat{X}}^{\left(j\right)}-X^{\left(j\right)}∥}_{2},$$

where $\hat{X}$ and *X* denote the predicted and ground-truth 3D joints, respectively. The second metric was reconstruction error, which is calculated after aligning the estimated 3D pose to the ground-truth 3D pose using the Procrustes method. In the case of reconstruction error, $\hat{X}$ in Equation (12) is replaced with 3D joints transformed using the Procrustes method. Following the typical protocol [1], reconstruction error was tested only on the front camera of S9 and S11 sampled every five frames. The last metric was the mean of the root position error (MRPE), which was first proposed in [10]. MRPE measures the Euclidean distance between the estimated and the ground-truth root joints in the camera coordinate system as follows:

(13) $$MRPE=∥{\hat{R}}_{cam}-R_{cam}{∥}_{2},$$

where ${\hat{R}}_{cam}$ and $R_{cam}$ denote the predicted and ground-truth root joints, respectively.

MuCo-3DHP and MuPoTS-3D datasets are 3D multi-person pose estimation datasets proposed in [20]. The training dataset, MuCo-3DHP, was generated by compositing the 3D single-person pose estimation dataset MPI-INF-3DHP [38]. The test dataset, MuPoTS-3D, contained 20 real-world scenes captured outdoors for up to three subjects and was annotated with ground-truth 3D poses. Following the previous work [10], we augmented the background of the MuCo-3DHP dataset using the MS-COCO [39] dataset. When training using the MuCO-3DHP dataset, we additionally used the in-the-wild 2D pose dataset, MS-COCO, for generalization, and each mini-batch consisted of half MuCo-3DHP and half MS-COCO data. For MS-COCO data, the indicator function of $L_{3D}$ and the weight for the absolute depth of $L_{root}$ were set to 0 because the MS-COCO dataset has no 3D annotation. We used three evaluation metrics for the MuPoTS-3D dataset. The first two evaluation metrics were the 3D percentage of correct keypoints (3DPCK${}_{rel}$) and the area under 3DPCK curve (AUC${}_{rel}$), for which we first aligned the root joints of the predicted and ground-truth 3D poses. Then, for 3DPCK${}_{rel}$, if the Euclidean distance between the predicted and ground-truth joints was within 15 cm, the predicted joint was considered correct. For AUC${}_{rel}$, 3DPCK was measured from various thresholds, and then the area under the 3DPCK curve was computed. The last evaluation metric, 3DPCK${}_{abs}$, was calculated in the camera coordinate system, wherein the predicted joint was considered correct if the predicted 3D joint lay within 15 cm from the ground-truth joint.

### 4.2. Comparison with the Baseline Method

We present comparison results with the top-down baseline method. We modified the method of [10] that estimates the 3D poses of multiple persons in the camera coordinate system in a top-down manner and adopted it as a baseline method because no other method could reconstruct the 3D shapes of multiple persons in the camera coordinate system. The model in [10] consists of PoseNet that predicts the root-relative 3D pose and RootNet that predicts the absolute 3D position of the root joint. PoseNet uses a volumetric heatmap representation and applies the soft-argmax operation to the volumetric heatmap to compute the 3D coordinates. We implemented a method that reconstructs the 3D shapes of multiple persons in the camera coordinate system in a top-down manner by replacing PoseNet, which predicts root-relative 3D pose, with the human mesh recovery (HMR) proposed in [1]. HMR extracts the convolutional features without removing the average pooling layer of the ResNet-50 backbone network and then predicts SMPL parameters through regression to reconstruct the 3D shape for a single person. The baseline method combines the outputs of RootNet and HMR to reconstruct the 3D shape finally in the camera coordinate system. We call the baseline method RootHMR. Similarly to the proposed method, the baseline method uses images of 448×448 size as input to the network.

Quantitative results. Table 1 and Table 2 show the quantitative comparison results of the proposed method and RootHMR on the Human3.6M dataset. RootHMR is a top-down method, which crops each person from the input image using the ground-truth bounding box information, resizes it to the size of the network input, and feeds it into a 3D reconstruction model for a single person. RootHMR performs a 3D human shape reconstruction robust to the human scale because it feeds the resized bounding box into the single-person shape reconstructor. However, the proposed method reconstructs the 3D shapes of multiple persons in a single-shot approach. The proposed method had higher MPJPE results than the top-down baseline method because it does not consider differences in the scale of multiple persons in the input image. As for the estimation of absolute depth, the proposed method uses the bounding box information predicted by BRP. Although the ground-truth bounding box was not used, the proposed method achieved MRPE results comparable with that of the baseline method.

Table 3 shows the quantitative results of the proposed method and RootHMR on the MuPoTS-3D dataset. For 3DPCK${}_{rel}$ and AUC${}_{rel}$ metrics, the proposed method had worse performance than RootHMR but achieved higher performance for 3DPCK${}_{abs}$. Unlike the Human3.6M dataset Kolotouros et al. [27] proposed a method that combines the feedforward regression step and the SMPLify-basedoptimization step into a loop structure to merge the advantages of a regression-based method and anoptimization-based method. In addition to the human body, methods for 3D reconstruction of thehuman hand and face have recently been proposed. The newly proposed method of Wu et al. [28] reconstructs the 3D shape of a human face based on unsupervised learning. All these deep learning-based methods reconstruct the 3D shape of a single person. The 3Dmulti-person shape reconstruction problem has been studied less than the single-person case. Zanfir et al. [9] reconstructed the 3D shapes of multiple people in a bottom-up fashion. They proposeda limb-scoring network to predict the connection likelihood between joints and groups the jointsusing binary integer programming. Their method is a bottom-up approach, but its disadvantage isthat it cannot process the multi-person 3D shape reconstruction task in real time because of the highcomputational complexity of the optimization using binary integer programming. In this paper, wepropose an end-to-end learning-based model for single-shot, 3D, multi-person shape reconstruction inthe camera coordinate system from a single RGB image.3. Proposed MethodsOur goal is to reconstrthat contains only single-person images in an indoor environment, the MuPoTS-3D dataset includes images of multiple persons in an outdoor environment, which makes absolute depth estimation relatively more difficult. We believe that the proposed single-shot bottom-up method enables more accurate prediction of absolute depth compared with the baseline method because global contextual information can be utilized more effectively by feeding the entire input image into the network.

**Figure 5:**
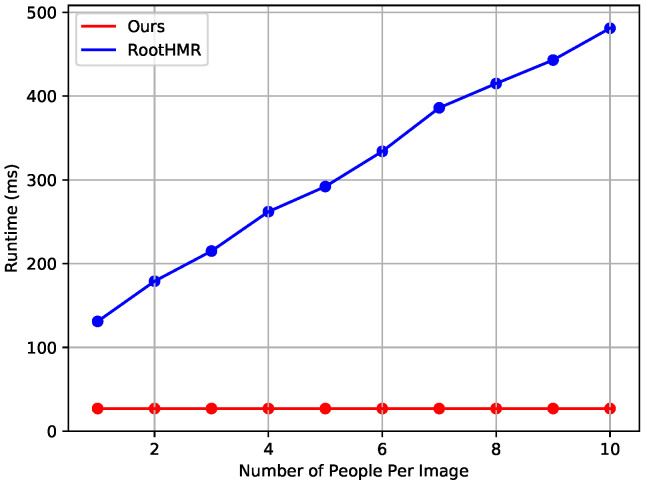
Runtime comparison with the baseline method.

Runtime measurement results. The runtime of the proposed network is constant regardless of the number of persons in the video because the proposed method reconstructs the 3D shapes of all persons in the image in a single step from the grid cells obtained by a single feedforward operation. By contrast, the top-down baseline method first uses a human detector to detect persons in an input RGB image, and then crops the detected area and feeds each into a single-person pose estimator. Therefore, in the top-down baseline method, the runtime increases as the number of persons detected in the image increases. In this experiment, the Mask R-CNN model with ResNeXt-101-32 backbone, which was pre-trainined using the MS-COCO dataset, was used as a human detector. Figure 5 shows the results of the runtime comparison with RootHMR. The proposed method had a constant runtime of about 27ms regardless of the number of persons, but the baseline method required additional runtime for the human detector, and runtime increased proportionally as the number of persons increased. Considering the real-time scenario, the baseline method does not process images in real time, but the proposed method processes images at about 37 fps and operates in real time.

Our experiments on runtime were performed under the assumption that the images were located in computer memory. We conducted additional tests to investigate runtime in a more realistic environment, including capturing images from a camera and pre-processing images. As a result of the experiment using a webcam, the proposed system reconstructed the 3D shapes of multiple persons at about 26 fps, which shows the high practicality of the proposed method and applicability to various areas.

### 4.3. Comparison with State-of-the-Art Methods

Table 4 shows the results of the proposed methods for the Human3.6M dataset and compares our method with the state-of-the-art methods that reconstruct the 3D human shape using the SMPL. State-of-the-art methods except our method focus on a single person, and the methods in [1,2,3,40] apply losses directly to SMPL parameters. Our method is a single-shot method of reconstructing the 3D human shapes of multiple persons, but it outperforms many state-of-the-art methods [3,24,41] that focus on a single person. Moreover, the proposed method can estimate the absolute position of multiple persons in the camera coordinate system, but other methods do not.

**Figure 6:**
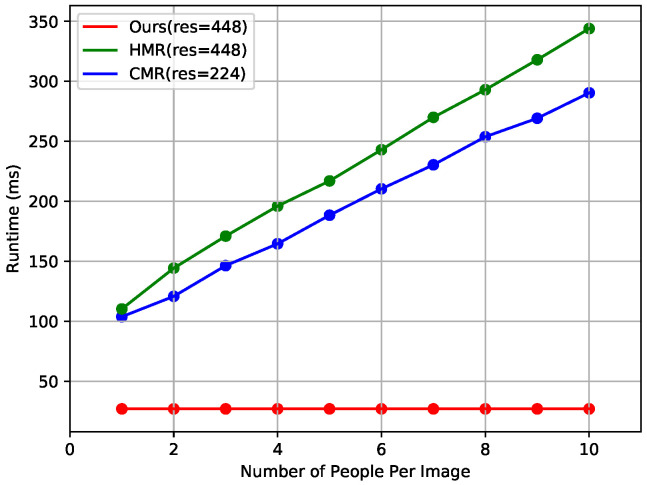
Runtime comparison with state-of-the-art methods. "res" denotes the resolution of the input image.

Figure 6 shows the results of runtime comparison with state-of-the-art methods that reconstruct the 3D human shape in a top-down fashion. We downloaded publicly available codes for HMR (https://github.com/MandyMo/pytorch_HMR) [1] and CMR (https://github.com/nkolot/GraphCMR) [40], which were run under the same conditions as the proposed method on our computer. HMR and CMR require additional runtime for human detection, and runtime increases proportionally as the number of persons in the image increases because each area cropped from the human detector is fed into the single-person shape reconstructor. By contrast, the proposed method has a constant runtime of about 27 ms regardless of the number of persons and outperforms state-of-the-art methods in terms of runtime.

## 5. Conclusions

In this paper, we propose an end-to-end learning-based model for single-shot 3D multi-person shape reconstruction based on the camera coordinate system from a single RGB image. Our network uses a grid-style representation and predicts information about the person in each grid cell to perform shape reconstruction in a single-shot manner. The network additionally predicts information about the bounding box, and the absolute depth of the root joint is calculated using the predicted bounding box information because our method assumes that bounding box information is not given as input. Finally, our network combines the root-relative 3D shape with the absolute position of the root joint to reconstruct the 3D shapes of multiple persons in the camera coordinate system. Experiments show that the proposed method achieves quantitatively better performance than many state-of-the-art methods. Moreover, we show through comparison with the baseline method that the proposed method achieves quantitatively comparable performance while outperforming it in terms of runtime. In future work, we plan to exploit video datasets to impose additional constraints on the human body structure or construct a sophisticated backbone network to obtain better image features for 3D shape reconstruction.

## Figures and Tables

**Table 1:** Mean per joint position error (MPJPE) comparison with the baseline method on the Human3.6M dataset. Smaller numbers denote better performance.

Methods	MPJPE ↓
RootHMR	80.8
Ours	92.8

**Table 2:** Mean of the root position error (MRPE) comparison with the baseline method on the Human3.6M dataset. Smaller numbers denote better performance.

Methods	MRPE${}_{x}$ ↓	MRPE${}_{y}$ ↓	MRPE${}_{z}$ ↓	MRPE ↓
RootHMR	27.5	35.9	93.7	115.3
Ours	22.8	21.0	115.5	126.1

**Table 3:** Comparison with the baseline method on the MuPoTS-3D dataset. Larger numbers denote better performance.

Methods	3DPCK${}_{\mathrm{rel}}$ ↑	AUC${}_{\mathrm{rel}}$ ↑	3DPCK${}_{\mathrm{abs}}$ ↑
RootHMR	68.2	31.5	17.4
Ours	51.1	22.8	19.1

**Table 4:** Reconstruction error comparison with state-of-the-art methods on the Human3.6M dataset. The numbers of the state-of-the-art methods were obtained from their original papers. Smaller numbers denote better performance.

Methods	Rec. Error ↓
Lassner et al. [41]	93.9
SMPLify [24]	82.3
Pavlakos et al. [3]	75.9
NBF [2]	59.9
HMR [1]	56.8
CMR [40]	50.1
Ours	65.9
